# The association between retraction of the torn rotator cuff and increasing expression of hypoxia inducible factor 1α and vascular endothelial growth factor expression: an immunohistological study

**DOI:** 10.1186/1471-2474-11-230

**Published:** 2010-10-08

**Authors:** Stefan Lakemeier, Johannes JA Reichelt, Thilo Patzer, Susanne Fuchs-Winkelmann, Juergen RJ Paletta, Markus D Schofer

**Affiliations:** 1 Department of Orthopedics and Rheumatology, University Hospital Marburg, Marburg, Germany

## Abstract

**Background:**

Differing levels of tendon retraction are found in full-thickness rotator cuff tears. The pathophysiology of tendon degeneration and retraction is unclear. Neoangiogenesis in tendon parenchyma indicates degeneration. Hypoxia inducible factor 1α (HIF) and vascular endothelial growth factor (VEGF) are important inducers of neoangiogenesis. Rotator cuff tendons rupture leads to fatty muscle infiltration (FI) and muscle atrophy (MA). The aim of this study is to clarify the relationship between HIF and VEGF expression, neoangiogenesis, FI, and MA in tendon retraction found in full-thickness rotator cuff tears.

**Methods:**

Rotator cuff tendon samples of 33 patients with full-thickness medium-sized rotator cuff tears were harvested during reconstructive surgery. The samples were dehydrated and paraffin embedded. For immunohistological determination of VEGF and HIF expression, sample slices were strained with VEGF and HIF antibody dilution. Vessel density and vessel size were determined after Masson-Goldner staining of sample slices. The extent of tendon retraction was determined intraoperatively according to Patte's classification. Patients were assigned to 4 categories based upon Patte tendon retraction grade, including one control group. FI and MA were measured on standardized preoperative shoulder MRI.

**Results:**

HIF and VEGF expression, FI, and MA were significantly higher in torn cuff samples compared with healthy tissue (p < 0.05). HIF and VEGF expression, and vessel density significantly increased with extent of tendon retraction (p < 0.05). A correlation between HIF/VEGF expression and FI and MA could be found (p < 0.05). There was no significant correlation between HIF/VEGF expression and neovascularity (p > 0.05)

**Conclusion:**

Tendon retraction in full-thickness medium-sized rotator cuff tears is characterized by neovascularity, increased VEGF/HIF expression, FI, and MA. VEGF expression and neovascularity may be effective monitoring tools to assess tendon degeneration.

## Background

Rotator cuff injury affects both the tendons and the associated muscles. Full thickness tears may result in muscle and tendon retraction, leading to a change in the angulation of muscle fibers and subsequent fatty infiltration (FI) [1]. FI was first observed by computed tomography (CT), which led to the five- stage1 classification system that is based upon fat-to-muscle ratio, one of the most important prognostic indicators after rotator cuff repair [1]. Furthermore, rotator cuff muscle atrophy (MA) is important in assessing treatment of rotator cuff tears [2-5]. Although these radiological parameters are good indicators of tendon degeneration, the histopathology of the degenerating rotator cuff are not as clear. Benson et al. proposed that hypoxic changes in the cuff contributes to loss of cells by apoptosis, which may be responsible for the development of tears [6]. These authors found different levels of hypoxia inducible factor 1α (HIF) in the damaged rotator cuff [7]. HIF is known to be an important upregulator of vascular endothelial growth factor (VEGF), which stimulates endothelial cells and vessels to invade hypovascularized tissue [8]. Pufe et al. and Peterson et al. found higher concentrations of VEGF in degenerative Achilles tendon as compared with healthy tissue [9,10]. Although the rotator cuff injury has been attributed to hypovascularity, there is some evidence that neovascularization also occurs [11]. Lewis et al. described increased neovascularity in patients with rotator cuff tendinopathy; increased HIF and VEGF expression in the diseased rotator cuff, but not in tendon retraction [12]. Furthermore, neovascularity has not been definitively proven histologically until this point.

The purpose of this study is to examine HIF and VEGF expression, and neovascularity in torn rotator cuff tissue from patients with medium size (3-5 cm, formally Bateman stage 3) full-thickness tears and tendon retraction, compared with healthy control samples. A second goal is to find an association between FI, MA and the amount of VEGF and HIF expression and neovascularity within both torn rotator cuffs.

## Methods

### Patients

39 patients (15 male, 24 female) was prospectively included in our study. Ethical approval was obtained from the ethics committee of the Medical Faculty at the University of Marburg, Germany. 33 patients had full-thickness rotator cuff tears which were refixtaed and closely repaired arthroscopically by double-row suture bridge technique performed by the senior author. Rotator cuff specimens were harvested from the margin of the tear site of the supraspinatus tendon during surgery. The control group consisted of six trauma patients with humeral head fractures; normal rotator cuff samples were harvested during open reduction and internal fixation from the supraspinatus tendon. Shoulder osteoarthritis was excluded by magnetic resonance imaging (MRI). Only patients with medium-sized full-thickness rotator cuff tears (tear size 3-5 cm) were included into this study. Tendon retraction was defined based on intraoperative findings using Patte's classification (stage 1: proximal stump close to enthesis, or bony insertion; stage 2: proximal stump at humeral head; stage 3: proximal stump at glenoid) [13]. Patients were assigned to 4 categories according to Patte stage and including one control group.

### Radiographic Assessment

All patients underwent preoperative shoulder MRI. FI was assessed according to the Goutallier-derived classification as adapted for use on oblique parasaggital T1-weighted fast-spin echo sequences [1,5]. The stages identified are: stage 0, normal muscle; stage 1, some fatty streaks; stage 2, manisfest fatty infiltration, but less than muscle; stage 3, as much fat asmuscle; and stage 4, more fat than muscle. FI was expressed for the supraspinatus muscle. Supraspinatus MA was measured on the most lateral image on which the scapular spine was in contact with the scapular body on oblique and saggital views as described by Thomazeau et al [4]. The cross-sectional area of the supraspinatus muscle and its fossa were digitally measured using a picture analyzing system (PACS, Agfa-Gevaert group, Mortsel, Belgium). Results were expressed as the standadized muscle area, defined by the cross-sectional area of the muscle to the cross-sectional area of the fossa [5,14].

### Specimen Preparation

Rotator cuff samples were immediately fixed in 4% formaldehyde for 24 hours, dehydrated in graded alcohol solution and cedarwood oil and embedded in paraffin. Sections were cut at 5 μm with a Leica microtome RM2055 (Bensheim, Germany) 40° stainless-steel knife. Histological standard staining was performed with a Masson-Goldner staining kit (Merck, Dramstadt Germany) according to manufacturer instructions.

### Histology

**Figure 1:**
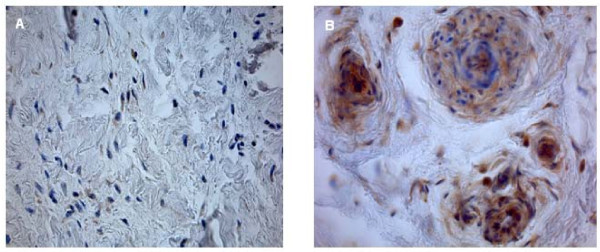
**Rotator cuff slices after straining with VEGF antibody (40× magnification)**. In the control group, VEGF expression is minimally observed (a); VEGF expression is high in group IV (b).

**Figure 2:**
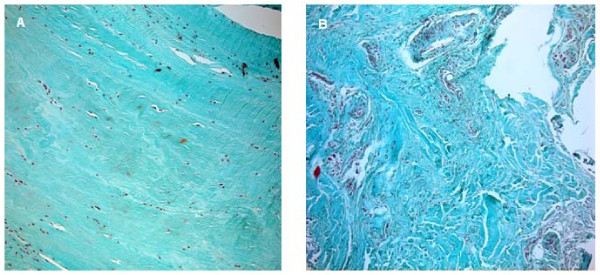
**group II (Patte I) (a) has low vessel density**. Group IV (Patte III) shows many visible vessels (b) (Rotator cuff slices, 10× magnification, Masson-Goldner staining).

**Figure 3:**
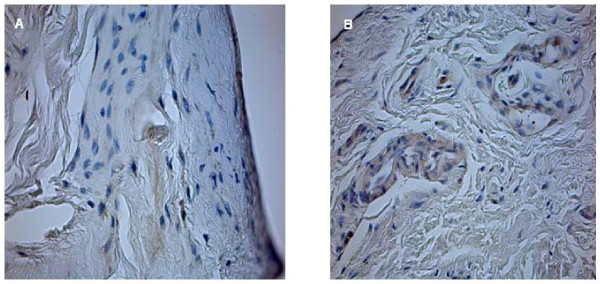
**Rotator cuff slices after straining with HIF antibody (40× magnification)**. Minimal HIF expression is detected in group I (control group) (a). Many HIF positive cells in group IV (Patte III) (b).

Histomorphometrical analysis was performed at a primary objective lens magnification of 5× using a Leica DM5000 and Quips analysis software (Leica Bensheim, Germany). In order to characterize the cells and to perform cell count, 40× objective lens magnification was used. For differential stained slices, for example Masson Goldner stained slices, a 10× objective lens magnification was used. Vessel number and size was determined by counting and measuring the vessels in three different areas of every specimen. Cell counting of immunohistological stained slices was performed by a 40× objective lens magnification. The percentage stands for the quotient of VEGF and HIF positive cells in relation to the total number of cells per slice. The percentage stands for the quotient of HIF or VEGF positive cells in relation to the total number of cells per slice. Figures 1, 2 and 3 show examples for different tendon sections and stainings.

### Immunochemistry

For immunohistological staining of HIF and VEGF, the slices were rehydrated and incubated in citrate buffer (pH 6) at 90°C over a period of 10 minutes. After blocking with normal horse serum, the slices were incubated overnight (4°C, humidified chamber) with a monoclonal antibody: VEGF clone VG1; MAB3734, rabbit, dilution 1:750, Millipore, Bilerica, USA. HIF clone H1alpha67, mouse, dilution 1:100, Dianova, Hamburg, Germany.

Immunostaining was performed using the labeled streptavidin-biotin method (Dako REAL Detection System Peroxidase/DAB+), the staining reaction being based on 3,3'-diaminobenzidine (DAB). The stained slides were rinsed with distilled water and stained for 15 seconds with hematoxylin and eosin as a counterstain. Finally, the sections were rinsed with water and treated with graduated density alcohol and xylol.

### Statistics

Analysis of variance (ANOVA) and a modified least-square difference (Bonferroni) test were used to evaluate the differences between the different groups. Data are reported as the mean ± standard error of measurements (SEM). Correlations between FI, MA, vessel count, VEGF and HIF were calculated according to Pearson's chi-square test. A p-value of less than 0.05 was considered significant.

## Results

### Patient and Tissue Characteristics

The supraspinatus muscle was injured in 100% (33/33) of patients, the infraspinatus muscle in 55% (18/33). There were no subscapularis or teres minor tears. Patient demographics (age, gender), mean HIF and VEGF expression, vessel density, vessel size, and FI and MA classification schemes are outlined in Additional file 1.

### Changes in Cross-Sectional Muscle Area

The fascial area was measured to be 212 ± 114 mm^2 ^in group I, 134 ± 98 mm^2 ^in group II, 142 ± 87 mm^2 ^in group III, and 120 ± 58 mm^2 ^in group IV. The bony area was 267 ± 97 mm^2 ^in group I, 245 ± 87 mm^2 ^in group II, 275 ± 55 mm^2 ^in group III, and 241 ± 57 mm^2 ^in group IV. Differences between groups I and II-IV were significant on facial area (p = 0.031). Other differences between muscle areas were not statistically significant.

### Fatty Infiltration

In the control group (group I), average FI was 0.5 (range 0-1); in group II (Patte stage 1), 2.3 (range 1-3); in group III (Patte stage 2), 2.7 (range 2-4); and group IV (Patte stage 3) 3.5 (range 2-4). The differences in FI measurements between group I and II-IV were significant (p = 0.018) but not between groups II and III. FI was significantly higher in group IV (Patte stage 3) than in groups II and III (Patte stage 2-3) (p = 0.036).

### HIF, VEGF Expression and Neovascularity

**Figure 4:**
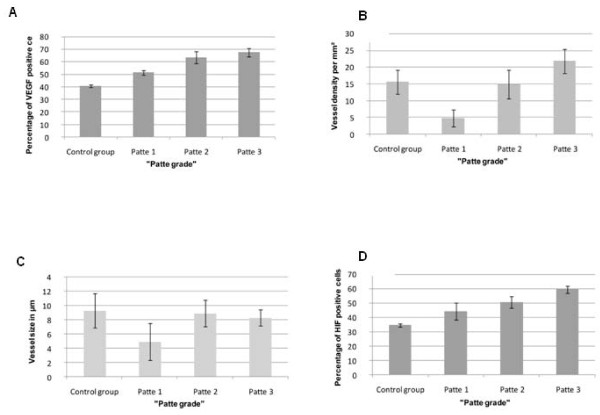
**VEGF expression (a), vessel density (vessels/mm^2^) (b), vessel size (μm) (c) and percentage of HIF expression (d) for the different groups**.

HIF and VEGF expression was significantly elevated in groups II-IV (Patte stage 1-3) in comparison with controls (group I) (p = 0.045, Figure 4a-d). Mean vessel density was significantly higher in group IV than in groups I, II and III (p = 0.011). There was no correlation between vessel size and vessel density in groups II-IV compared with controls. Mean HIF and VEGF expression and vessel density is outlined in Additional file 1.

### Correlations

We found a significant correlation between FI, MA, and HIF and VEGF expression in medium sized rotator cuff tears between groups II-IV and controls. There was a significant correlation between vessel density, FI, and fascial MA in group 4 (p = 0.027). Other differences and correlations were not statistically significant.

## Discussion

We aimed to clarity the relationship between VEGF/HIF expression and neovascularization and tendon retraction in full-thickness rotator cuff injuries. We showed that HIF and VEGF are upregulated in full-thickness rotator cuff tears (highest in group IV, Patte stage1 III), leading to higher vessel density while angiogenetic peptides are not detected in healthy tendon. In the torn rotator cuff, vessel density was lower than in our control group but increased with worsening tendon retraction. Our results confirm the findings of Matthews at al., who reported smaller numbers of vessels in massive tears compared to healthy controls [15]. However, vessel density was not explored in that work; for the first time, we have shown increasing vessel density correlating with retraction. This could be due to high VEGF levels, although we did not find this in our patient population. This may be due to the small number of patients in groups I and II.

While VEGF mediated angiogenesis contributes to the repair and remodeling of degenerated tendons, invasion of endothelial cells may also weaken the mechanical stability of the tendon [8]. We assume that increasing vessel density weakens the tendons and leads to the accretive nature of tendon retraction. This has been supported by the recently published theoretical model of Peers et al, showing that chronic tendon loading causes mechanical trauma with multiple microruptures of tendon microvasculature [16]. These microruptures initiate a VEGF-mediated cascade of vascular remodeling that becomes chronically pathologic. Hypervasularity has been demonstrated in degenerated human and rabbit Achilles tendons, in diseased patellar and long head biceps tendons [17-20]. Chronic tendon pathology appears to be a highly active process of ongoing neovascularisation [21]. Why this process does not lead to tissue repair, but to pain and chronic disease, is unknown.

Primary reconstruction of massive rotator cuff tears with minimal retraction is currently recommended in clinical practice; more extensive reconstruction with muscle transfers and prostheses are used in cases of moderate to severe retraction [22-24]. Our findings present histological backing for these guidelines and imply a potential benefit to treating patients with partial thickness tears more aggressively, in order to prevent complications of injury progression.

FI and MA of the torn rotator cuff correlate with the duration and size of the tear [3,4,25]. MA and FI levels could be used as outcome predictors for surgically treated injuries [26]. There is accumulating evidence that these parameters correlate closely with tendon degeneration. While age has been previously shown to correlate with FI and MA, we did not confirm this in our study. This may be due to the consistency of age among our patients groups. We demonstrated a correlation between FI and MA and VEGF/HIF expression. Therefore, VEGF and HIF expression as well as neovascularity may be used as a monitoring tool in the clinical setting, for the level of tendon degeneration that occurs during tendon degeneration. Neovascularization has been previously described in massive rotator cuff tears as compared to smaller tears of smaller size [27]. Neovascularization and lipid accumulation may be an indication of enhanced aerobic metabolism in the degenerated muscle. Further histological studies are required to investigate the mechanism of this finding; HIF and VEGF upregulation may help to explain these observations.

HIF, which is induced by cell hypoxia, is one of the most important regulators of VEGF gene expression [28]. The original function of VEGF as a strong inducer of embryonic vasculogenesis was shown in knockout mice [29]. VEGF plays a pivotal role in tumor angiogenesis (e.g. glioblastomas), as well as other disease states (diabetic retinopathy, age-related macular degeneration or rheumatoid arthritis) [28]. While the role of VEGF in tendon degeneration is largely unknown, the process is thought to occur in areas of poor perfusion. Rahburn and Macnab identified a hypovascularized zone in the rotator cuff that coincides with the most common location for tears [30]. In contrast, we found hypervascularisation within our samples. Our findings are supported by studies showing increased Doppler flow in diseased rotator cuffs, likely reflecting a neovascularization process [12,31].

The etiology of rotator cuff tears is still a matter of debate. Some authors ascribe mechanical damage to subacromial impingement, while others suspect a primary process of tendon degeneration [11]. On the basis of ultrasound findings, Gohlke found that patients with degenerative full-thickness rotator cuff tears and significant retraction were older [32]. In a recent literature review, Nho et al. conclude that hypovascularity is a minimal contributor to cuff tears, which supports our finding that hypervascularisation occurs late in the course of cuff tendinopathy [33]. Since HIF induction was initially described in conditions of decreased oxygen partial pressure, the majority of studies on HIF regulation have addressed hypoxic conditions [34]. However, there is evidence that HIF can also be induced under normal conditions by growth factors, hormones or nitric oxide [35].

Cyclic loading is as important inducer of HIF expression. Benson et al. showed HIF levels to be high in mild impingement, small, medium, and large tears, but reduced in massive tears. We assume that complete tendon rupture reduces mechanical cyclic loading, resulting in reduced HIF expression. If hypoxia underlies HIF expression, this contradicts Matthews's results describing the poorest blood supply in massive tears [15]. Biomechanical studies of Andarawis-Puri and Perry found out that cyclic tendon loading attenuates as retraction increases [36,37]. In our findings, HIF expression significantly increases with rising Patte grade. Therefore, it remains unclear whether hypoxia or cyclic tendon loading is the underlying reason for HIF and VEGF expression.

Our study has several limitations. Our control group is comparatively small; tumor patients were excluded from the study due to their requirement for prosthesis implantation or upper limb amputation. Therefore, we elected to include trauma patients of matchable age as controls (difficult due to the age discrepancy in this population as compared with the other groups and the reason for our low numbers).

Full-thickness rotator cuff tears may develop into cuff arthropathy and be associated with osteoarthritis of the shoulder [38]. Osteoarthritis was not measured, but may act as a confounder for VEGF and HIF expression. This bias was addressed by limiting our subjects to patients with medium sized tears (3-5 cm; unclassified due to lack of international consensus). Extent of tendon retraction was the only fluctuating parameter.

## Conclusion

We demonstrated a correlation between vessel density, VEGF, and HIF expression as well as FI and MA in full-thickness medium-sized rotator cuff tears and tendon retraction. Neovascularization in cuff and tendon tissues is mediated by VEGF. Increasing vessel density accelerates tendon degeneration by weakening its underlying structure. VEGF expression and neovascularity could be used in the clinical setting to monitor tendon degeneration in patients with rotator cuff injuries.

## Competing interests

The authors declare that they have no competing interests.

## Authors' contributions

SL was the main composer of the manuscript; JJAR and JRJP performed histological and immunohistological testing. JRJP performed the statistical analysis and together with SL the radiographic assessments. SFW and TP conceived of the study, participated in its design and coordination, and helped to draft the manuscript. MDS designed the study, performed the surgeries, and obtained the tendon samples. All authors read and approved the final version of the manuscript.

## Pre-publication history

The pre-publication history for this paper can be accessed here:

http://www.biomedcentral.com/1471-2474/11/230/prepub

## Supplementary Material

Additional file 1 **Patient demographics with cuff tendon retraction classification, mean values for vessel density, vessel size, mean HIF and VEGF expression**. Click here for file
