# Supplementary material for: The association between retraction of the torn rotator cuff and increasing expression of hypoxia inducible factor 1α and vascular endothelial growth factor expression: an immunohistological study
Source: BMC Musculoskelet Disord. 2010 Oct 8;11:230. doi: 10.1186/1471-2474-11-230 (PMC2958987; doi:10.1186/1471-2474-11-230)
Supplement: Additional file 1 — Patient demographics with cuff tendon retraction classification, mean values for vessel density, vessel size, mean HIF and VEGF expression. [file 1471-2474-11-230-S1.RTF]

Diagnosis	Number of patients	Number of patients male	Number of patients female	Mean age
(years)	Mean vessel density (vessels/cm²) ± standard error	Mean vessel size
(µm) ± standard error	Mean HIF expression (percentage of positive cells) ± standard error	Mean VEGF expression
(percentage of positive cells) ± standard error	
Control group (group I)	6	3	3	56 (47-69)	15.51 ± 3.55	9.31 ± 2.41	34.42 ± 1.15	40.61 ± 1.15	
Patte grade 1
 (group II)	6	0	6	 61 (55-68)	4.75 ± 5.52	4.93 ± 2.62	44.14 ± 6.01	51.30 ± 1.91	
Patte grade 2 
(group III)	10	4	6	65 (55-75)	14.82 ± 4.33	8.92 ± 1.85	50.57 ± 3.97	63.35 ± 4.70	
Patte grade 3 
(group IV)	17	8	9	69 (51-79)	21.75 ± 3.60	8.32 ± 1.12	59.44 ± 2.39	67.49 ± 3.45	

Additional file 1: Overview of the included patients, the classification of their cuff tendon retraction, mean values for vessel density, vessel size, mean HIF and VEGF expression.
